# The Theory and Practice of Counter-Irritation

**Published:** 1896-03

**Authors:** 


**The Theory and Practice of Counter-Irritation.** By H. Cameron Gillies, M.D. Pp. xii., 236. London: Macmillan & Co. 1895.

Counter-irritation has of late fallen rather into disuse, and mainly on theoretical grounds. "The theorist is always the pioneer of scientific progress," and hence the need for a review of our position with regard to counter-irritation. No one applies a remedy without some theory as the motive and justification of his action, and no one ever gave up a theory because of discouraging results. Some people dislike theories, and profess to be superior to them: they act, and despise thought; but few of us are able to act efficiently without theoretical supports.

The author reviews at considerable length the history of the idea; but he does not wish to be esteemed an apostle of counter-irritation: he expresses himself "more in sympathy with those who hold that 'the Devil himself, old Beelzebub, is nothing but a great Cantharid' (Baynard) than with those who would commend twelve consecutive applications of actual cautery for the cure of sciatica (Peter)." He combats many of the older interpretations such as were implied in words like revulsion, derivation, evacuation, vicarious action, and metastasis: these "should be allowed to fall out of medical terminology." And then he enunciates his own theory, which is based upon the idea that inflammation is a reparative process, that it is the name given to a process of physiological activity which is directed to the repair of an injury; and hence it follows that "whatever good comes by the use of counter-irritants is because, by their irritant effects, they stimulate the activity of the tissues of the part to which they are applied and accelerate the blood supply thereto, so increasing nutrition or repair as the need may be." He maintains that the essential action of a counter-irritant is to quicken the circulation in the part to which it is applied: and on this fact depend its uses in quickening the process of repair, in giving relief to pain, in promoting absorption and removal of pathological products, in aiding and accelerating the natural effort to throw off dead tissues, in removing a state of passive congestion, in rousing from a state of unconsciousness, and in drawing poisonous products from the tissues. Clearly the book is one to be read and digested.

